# Drivers of Antimicrobial Use Practices among Tennessee Dairy Cattle Producers

**DOI:** 10.1155/2018/1836836

**Published:** 2018-12-27

**Authors:** John E. Ekakoro, Marc Caldwell, Elizabeth B. Strand, Chika C. Okafor

**Affiliations:** ^1^Department of Biomedical and Diagnostic Sciences, College of Veterinary Medicine, University of Tennessee, 2407 River Drive, Knoxville, TN 37996, USA; ^2^Department of Large Animal Clinical Sciences, College of Veterinary Medicine, University of Tennessee, 2407 River Drive, Knoxville, TN 37996, USA

## Abstract

Nonjudicious antimicrobial use (AMU) and inadequate antimicrobial stewardship are known modifiable factors driving the occurrence of antimicrobial resistance (AMR). A mixed methods approach using a combination of focus groups and survey questionnaires was used to explore the AMU practices of Tennessee (TN) dairy cattle producers. Specifically, the objectives of the study were to determine the following: (1) the most common drivers for using antimicrobials, (2) perceived alternatives to antimicrobials, (3) knowledge of and perceptions regarding AMR, (4) and the appropriate avenues for receiving information on prudent AMU. Two focus groups were conducted, one in July 2017 and the other in March 2018. The questionnaire was simultaneously made available to participants both in print form and online from January 26, 2018, through May 11, 2018. Twenty-three dairy producers participated in the focus groups and 45 responded to the survey. Eight (18.6%) producers never used bacterial culture and sensitivity testing (C/S) to select antimicrobials, more than half (25 producers (58.1%)) sometimes used C/S, four (9.3%) used C/S about half the time, five (11.6%) most of the time, and one (2.3%) always used C/S. The most common drivers for using antimicrobials were disease and animal welfare, pathogen surveillance, economic factors, veterinarian recommendation, producer's experience and judgment, drug attributes, and the Veterinary Feed Directive. Good management practices, vaccination, use of immunomodulatory products, and use of appropriate technology for early disease detection were considered alternatives to AMU. Four (9.1%) dairy producers were very concerned about AMR, 27 (61.4%) moderately concerned, and 10 (22.7%) not concerned. The veterinarian was considered to be a trusted source of information on prudent AMU. Use of C/S test results for antimicrobial selection is widespread among TN dairy producers. There is a need to popularize/promote selective dry cow therapy among TN dairy producers.

## 1. Introduction

Antimicrobial resistance (AMR) is now recognized as a major global health problem [1, 2]. Nonjudicious antimicrobial use (AMU) and inadequate antimicrobial stewardship (AMS) are known modifiable factors driving the occurrence of AMR [3]. The public health threat of AMR has led to increased societal pressure to limit AMU in food animals [4].

To prevent potential public health consequences of AMR, many countries have instituted measures to reduce and minimize AMU in food animals [3]. These measures are based on the precautionary principle, since there is currently no robust evidence on the public health impacts of AMU in food animals on AMR in human pathogens [3]. The precautionary principle of public health recommends the adoption of preventive measures in the face of uncertainty and exploring various alternatives to potential threats to public health [5]. Currently, it is not possible to quantify the risk of the zoonotic transmission of resistant bacteria from livestock to humans. Recent systematic reviews showed that although some primary studies suggested evidence of AMR transmission from and between food animals and humans, a large proportion did not provide evidence supporting such transmission [6–8]. Recent studies have shown that indiscriminate use of antimicrobials for both therapeutic and nontherapeutic purposes in animals leads to propagation and shedding of substantial amounts of AMR microorganisms [3, 9].

The World Health Organization (WHO) recommends complete restriction of AMU in food animals for growth promotion and for disease prevention, as well as a reduction in the overall use of medically important antimicrobials in food animals [1]. Beginning January 1, 2017, the United States (U.S.) Food and Drug Administration (FDA) implemented the Veterinary Feed Directive (VFD), aimed at facilitating the judicious use of medically important antimicrobials in food producing animals. The VFD authorizes the use of medically important antimicrobials in feed and water for therapeutic purposes, under the supervision of a licensed veterinarian. For policy interventions such as the VFD to be effective, factors that inform and influence or drive producer behavior in relation to AMU need to be addressed because producers consistently base their decisions and actions on a complex system of core values and knowledge.

In the Netherlands, implementation of the farm health plan and the farm treatment plan is obligatory for all farmers [10], must be developed in a collaboration between the producer and the herd veterinarian, and must be evaluated annually [11]. The independent Netherlands Veterinary Medicines Authority (SDa) collects and reports AMU and prescription data from all individual Dutch farms and veterinarians and high antimicrobial users and prescribers could be subjected to disciplinary sanctions by the quality assurance systems [12]. In the U.S., there are no legal or quality assurance scheme requirements for the collection of AMU data by dairy farmers. However, the FDA recommends establishment of written protocols for any AMU on individual farms in collaboration with the herd veterinarian [13].

Previous studies among dairy farmers identified veterinary advice, the producer's personal on-farm experience, disease occurrence, animal welfare, and the drug withdrawal period as primary factors driving choice and use of antimicrobials [14–16]. To date, however, there has been very limited investigation into the drivers of AMU practices of cattle producers in the US. No previous study to our knowledge has explored the drivers of AMU among Tennessee (TN) dairy cattle producers.

In this study, our aim was to use a combination of focus groups and survey questionnaires to explore the AMU practices of TN dairy cattle producers. Specifically, the objectives of the study were to determine the following: (1) the most common drivers for using antimicrobials, (2) perceived alternatives to antimicrobials, (3) knowledge of and perceptions regarding AMR, and (4) the appropriate avenues and formats for receiving information on prudent AMU. These findings should optimize the efforts under which targeted campaigns for nationwide AMS are applied in US dairy production.

## 2. Materials and Methods

### 2.1. Study Design

This was a mixed methods study using a combination of focus groups and survey questionnaires. To aid in the triangulation between the qualitative and quantitative data, preliminary findings from one focus group were used in the development of the survey questionnaire. The University of Tennessee Institutional Review Board for the Protection of Human Subjects in Research reviewed and approved both the qualitative (Protocol number: UTK IRB-17-03702-XP) and the quantitative (Protocol number: UTK IRB-17- 03884-XP) parts of this study.

### 2.2. Qualitative Methodology

#### 2.2.1. Focus Group Design, Structure, and Procedure

We conducted two dairy producer focus groups in middle TN and east TN in July 2017 and March 2018 respectively, and the participants were purposively selected. The middle TN focus group (focus group 1) was conducted with dairy producers attending an annual dairy producer meeting and was held at a local restaurant. Fourteen people attended this annual dairy producer meeting (12 of whom actively participated in the discussions). Participants in the east TN focus group (focus group 2) were recruited from dairy producers attending a master dairy training meeting held at a county extension center. Of the approximately 35 producers who attended this master dairy training session, 11 volunteered to participate. Each focus group meeting lasted approximately 60 minutes. Each participant was given an informed consent form with an overview of the study and a signed consent was obtained before participating in the focus group discussion. Participants could opt out of the focus groups at any time. A meal was provided to all invited participants irrespective of their active participation.

We used a semistructured interview guide consisting of 11 open-ended questions designed to address the study objectives (see file S1 in the supplementary materials for the interview guide). We assigned each participant an identity number to maintain anonymity. These identity numbers were used throughout the discussion and participants announced these numbers before speaking. The two focus groups were moderated by one of the authors (EBS). Three members of the research team (JEE, MC, and CCO) took hand written notes of any key points, provided clarifications to questions, and asked follow-up questions when necessary. We could not determine if data saturation was reached during the second focus group discussion. Each focus group discussion was video-recorded and later transcribed verbatim by a professional transcription service provider for thematic analysis.

#### 2.2.2. Data Analysis

We analyzed the transcripts using a data analysis software (NVivo qualitative data analysis Software; QSR International Pty Ltd. Version 12, 2018). Thematic analysis was performed using a recursive six-phase approach (familiarization with the data, generation of initial codes, search for themes, review of themes, definition and naming of themes, and report production) as described previously [17]. To familiarize themselves with the data, each member of the team (JEE, MC, EBS, and CCO) read both transcripts. The percent of word similarity between the 2 focus groups was assessed using Jaccard's coefficient. A master project with the two transcripts uploaded was developed by the primary author (JEE) and distributed to the other authors for individual coding. An inductive approach was used to develop a coding frame (each author created independent nodes). Upon completion of the individual coding, the primary author (JEE) imported the other team members' coded data into the master project and examined if the themes from the individual coding were related to the coded extracts in all the data transcripts. The degree of agreement in the data coding among the coders (JEE, MC, EBS, and CCO) was determined in NVivo using percent agreement. Results of the independent coding was reviewed and harmonized by the research team.

### 2.3. Quantitative Methodology

#### 2.3.1. Study Design and Administration of Survey

A survey questionnaire consisting of a section for dairy producers and another for beef producers was developed and evaluated by two professionals with expertise in AMU to ensure all critical issues were identified and covered (see file S2 in the supplementary materials for the survey questionnaire). Dairy producers completed only the dairy section of the questionnaire. Preliminary findings from focus group 1 were used in the development of the questionnaire. The 56 survey questions targeted the producer's demographics and their AMU practices, factors driving producer's choice of antimicrobials, and perceptions, opinions, and concerns about AMU and AMR in cattle production.

The targeted producer demographic information included age, sex (male versus female), level of education, herd size, whether raised on a livestock farm or not, and number of years in cattle farming. This demographic information was our explanatory variables of interest. Our main outcome of interest was the producers' degree of concern about antimicrobial resistant infections in cattle. Also, the association between level of education and producer's perception of antimicrobial label instructions was of interest. Three-point scales and ordinal Likert scales were used to capture participant responses to questions related to AMU practices, factors driving choice of antimicrobials, and perceptions, opinions and concerns about AMU and AMR in cattle production.

The questionnaire was simultaneously made available to participants both in print form and online. Participants who completed the print survey were requested not to complete the online survey and vice versa in the informed consent statement. The on-line version of the survey was housed in a survey software (Qualtrics software, Provo, UT) and was adapted for computer, tablets, and cell phone responses. The anonymize function in the Qualtrics software was optimized, so responses were not attached to any personal identifiers. During an annual dairy producer meeting in January 2018, producers were notified about the online survey option in order to increase the response rate. Subsequently, an email invitation to take the survey was sent out to all the 87 dairy producers on the University of Tennessee Animal Science department email list. The printed questionnaire was distributed to producers attending dairy producer meetings and master dairy training meetings across TN. Completed printed questionnaires were returned to the investigators or mailed to the last author.

Both the printed survey and online survey remained open from January 26, 2018, through May 11, 2018. Participation in the survey was voluntary and the survey targeted all dairy producers in the state (the estimated number of dairy producers in TN as of 2017 was 300) [18]. To further increase the response rate, follow-up email reminders were sent to nonrespondents of the online survey every two weeks. All participants were invited to participate in a $10 gift card raffle taken at the end of the survey and the winners were randomly selected. Eligibility to participate in the raffle was not contingent upon survey completion.

#### 2.3.2. Statistical Analysis

A commercial statistical software (SAS, version 9.4, SAS Institute Inc, Cary, NC) was used to complete descriptive and univariable inferential analyses. Descriptive statistics (frequencies and proportions) were used to summarize the data. Responses on the Likert scales were visualized using stacked bar charts created in another commercial software (Tableau software, version 8.2, Seattle, WA). No corrections were made to missing data.

Univariable analyses (ordinal models with PROC LOGISTIC) were performed to test for associations between the captured demographic information and producers' degree of concern about antimicrobial resistant infections in cattle (our primary outcome of interest). Model fit was assessed using the score test for the proportional odds assumption, deviance, and Pearson goodness-of-fit statistics. Also, binary logistic regression was used to test the association between level of education and producer's perception of antimicrobial label instructions. For the univariable analysis, level of education was reclassified into two categories, high school/vocational or ≥ college, while herd size was reclassified into ≤ 150 or ≥ 150 dairy cattle. The 95% confidence intervals were used to test significant associations. Values of P < 0.05 were considered statistically significant. Multivariable analyses were not performed because meaningful multivariable analysis was deemed to be untenable based on findings from the univariable analyses.

## 3. Results

### 3.1. Focus Group Participant Characteristics

A total of 23 dairy producers actively participated in the two focus groups. Focus group 1 had one female and 11 male participants, while focus group 2 had two females and nine male participants. The reported milking herd size per producer ranged from approximately 40 to 1,100 dairy cattle. There was no participant that self-identified as an organic dairy producer.

The responses from the 2 focus groups were 31.2% similar (Jaccard's similarity index = 0.312). This Jaccard's similarity index provided evidence that there was diversity among participants. Percent agreement (in coding) between each pair of coders was > 80%. The results from the focus group discussions are presented as guided by the consolidated criteria for reporting qualitative studies (COREQ): 32-item checklist (see file S3 in the supplementary materials for the COREQ checklist).

### 3.2. Survey Participant Characteristics and Self-Reported AMU Practices

Of the estimated 300 dairy cattle producers in the state, a total of 45 participated in the survey. Overall, the estimated survey response rate was 15%. The majority of respondents provided complete responses for most questions, except for a few cases where some respondents left some questions unanswered. Of the 45 dairy participants, 40 completed the print survey, while only five completed the online version. Thirty-nine (39) provided their gender: 31 males and seven females. One of these respondents preferred not to report their gender. The demographic information of the survey respondents is presented in Table 1. Majority of the participants mentioned that they kept up-to-date written records on antimicrobial purchases and did not practice extra-label AMU (Table 2).

### 3.3. Objective 1: Drivers of AMU Practices

The major themes identified as drivers of AMU in the focus groups were: (a) disease and animal welfare; (b) pathogen surveillance; (c) economic factors; (d) veterinarian recommendation; (e) producer's experience, and judgment; (f) drug attributes; and (g) the VFD. A detailed presentation of these themes from the focus groups and other survey findings salient to this objective are given below.

*(1a) Disease and Animal Welfare*. The decision to use antimicrobials by dairy farmers was influenced by the presence of early signs of disease, such as high rectal temperatures, droopy ears, and teary eyes. Mastitis was commonly mentioned as the reason for using antimicrobials. Producers believed it was their duty to ensure the welfare of their cattle through treatment with antimicrobials.

… if she's running a temperature, we try to get drugs in her pretty quick. … [No. 8, focus group 2].

… We treat all of our sick cows with antibiotics. We like to use some tetracycline in our calves to combat lots of things … [No. 3, focus group 2].

Among survey respondents, mastitis (n = 21), respiratory infections/pneumonia (n = 4), and lameness/hoof problems (n = 2) were mentioned as the most common diseases/conditions for which antimicrobials were used. Other diseases/conditions mentioned by survey respondents included enteric problems/scours (n = 1) and infectious bovine keratoconjunctivitis (n = 1). The most commonly used antimicrobial drugs mentioned by the survey participants belonged to cephalosporins (n = 13), tetracyclines (n = 7), penicillins (n = 3), and amphenicols (n = 1) antimicrobial classes. Ceftiofur (n = 10), cephapirin (n = 3), long acting oxytetracycline preparations (n = 5), and florfenicol (n = 1) were the most commonly mentioned individual antimicrobials used. These individual antimicrobials were often mentioned using their proprietary names.

*(1b) Pathogen Surveillance*. A section of focus group respondents self-reported that they used culture and sensitivity test results for on-farm pathogen surveillance. This use of culture and sensitivity testing influenced AMU in some dairy farms and reportedly led to reduced AMU.

…We recently started plating mastitis cows. That's been a big deal whether or not because before we would just treat anybody who got mastitis. And now we actually not 100 percent know the bug. But we know what group it's in. So that's kind of cut down on our antibiotic use as far as mastitis goes… [No.12, focus group 1].

…I've sent cultures[samples] off to university. Nine times out of ten, it's a form of e-coli. And he'll [the veterinarian] give you the drugs to take care of it… Once that's stopped to kill that bacteria, these drugs [do] not work no more… [No.4, focus group 2]

For producers who completed the survey questionnaire, the results of their self-reported use of bacterial cultures to determine the cause of disease on the farms, and their use of culture and sensitivity testing (C/S) to select antimicrobials are presented in Table 3.

*(1c) Economic Factors*. In the focus groups, the economic value of the animal was commonly mentioned to be an important driver of AMU. Animals perceived to be worth treating with antimicrobials were treated, while those perceived not worth treatment were culled and replaced by healthy stock.

… We started looking at cattle a lot closer. If she's actually worth the treatment? Or is it better just to [inaudible] and ship them down the road? I have kind of stressed that real hard amongst the employees. Before you treat, come to us; let's see is she worth it?... [No.5, focus group 1].

… Really, the history of the cow. If that cow is worth putting antibiotics in, calling the vet or whatever – we've sent some to slaughter because of her history. She's just not [worth treating] – and her genetics, too – she's carrying a good heifer cow or whatever, we look at that also… [No.9, focus group 2].

Among survey questionnaire respondents, four (10%) strongly agreed with the statement “profitability of your operation is an important factor influencing your decision to use antibiotics on your cattle,” 20 (50%) agreed with this statement, 10 (25%) neither disagreed nor agreed, four (10%) disagreed, and two producers (5%) strongly disagreed.

*(1ci) Lactation Stage and the Dry Period*. The stage of lactation (early lactation or late lactation) as well as the dry period influenced AMU practices of dairy producers.

…I mean, stage of lactation is probably first [determinant of antimicrobial use]... [No.10, focus group 1].

… [Animals are treated with antimicrobials] depending on dry cow or freshing cow or just depending on what stage of lactation they've come through… [No.6, focus group 2].

Some focus group participants reported using blanket dry cow therapy (intramammary antimicrobials are administered to all quarters of all cows in the farm at the end of lactation) at their farms to minimize the economic losses associated with intramammary infections, while others indicated that they do not use blanket dry cow therapy, but rather utilized selective dry cow therapy (cows receive antimicrobial treatment at the end of lactation only based on evaluation of the infection status of the cow or quarter. Only cows infected in one or more quarters are treated with intramammary antimicrobials in all quarters at dry off). In focus group 2, cessation of blanket dry cow therapy was associated with an increase in somatic cell counts.

…One thing that hasn't been mentioned is dry cow therapy, which is pretty much blanket treatment at our farms. [No.12, focus group 1].

…I was told by someone else to not [do] blanket dry treatment because I'm seasonal. So, I have to do [selective dry cow] treatment… [No.6, focus group 1].

…This is the first year that I didn't do that [blanket dry cow therapy]. And I've had more somatic cell count problems than I've ever had… [No.6, focus group 2].

*(1d) Veterinarian Recommendation*. For some producers with access to a veterinarian, veterinary recommendations influenced their AMU. However, others mentioned that the veterinarians they consulted had limited knowledge of dairy cattle restrictions. Cost was an additional barrier to seeking veterinary assistance.

…. We follow veterinarian recommendations as well.…. [No. 5, focus group 1].

… We have access to a group of veterinarians about an hour, 45 minutes away. They deal mainly with beef cattle on the large animal side. They know very little about dairy produce restrictions and that sort of thing. Like somebody said earlier, they ask me what we should use? When you get the bill, it kind of hurts your feelings.... [No.13, focus group 1].

*(1e) Producer's Experience and Judgment*. Most producers mentioned that they relied on their own experience, knowledge, and judgment when deciding to use antimicrobials in their cattle. This helped them reduce costs, such as veterinary fees, and helped them handle emergency cases in the event the veterinarian delayed. Furthermore, because producers are used to working with cattle on a daily basis, some dairy farmers believed they knew more about food animal issues compared to some veterinarians not used to working with food animals.

… Our vet lives over an hour away. So, if you have something that's an emergency, you still have to wait for him. In my experience, what happened with us was I just learned to do everything myself. So, they sort of worked their self out of a job. … [No. 12, focus group 1].

(*1f) Drug Attributes*. Perceived efficacy of the antimicrobial medicines, cost of antimicrobials, and the antimicrobial drug withdrawal times were mentioned as key factors influencing choice of antimicrobial drugs. Drugs perceived to be highly efficacious were preferred, while drugs with short withdrawal times were also preferred. It was mentioned that because some antimicrobials are very expensive, producers preferred highly efficacious products to avoid the additional costs of repeat treatments associated with treatment failure.

… Most important is an antibiotic that we use actually take care of the problem with one – not necessarily the same dose but one round of antibiotics. The problem's gone, and it doesn't return. If you go one round of antibiotics and the cow is fine and she's straightened up, and then two weeks later, she's got to get it again, that's not a good result from your antibiotics. We want one round to make sure it's all done; that problem's over with… [No.6, focus group 1].

Among questionnaire respondents, fifteen (37.5%) agreed with the statement “Aggressive marketing of antibiotics by pharmaceutical companies greatly influences producers' use of antibiotics,” 19 (47.5%) neither disagreed nor agreed with this statement, five (12.5%) disagreed, and one (2.5%) strongly disagreed with this statement. However, in the focus group discussions, marketing pressure from veterinary pharmaceutical company representatives was not identified as a driver of AMU.

*(1g) The VFD*. The VFD was believed to be driving the increase in the therapeutic use of antimicrobials, especially in calves, because it has restricted access to in-feed antimicrobials for disease prevention. Producers gave an example of Aureo S 700®, an in-feed antimicrobial preparation that was previously easily accessible to producers and now is restricted to use by or on the order of a licensed veterinarian. This restricted access to in-feed antimicrobials by federal law was reported to be leading to increased use of injectable antimicrobials by producers.

…We used it [aureomycin S 700] during winter stress times when it would get really cold. We would use it as a preventative thing. So, now [with the VFD] we doctor with something else once they get sick rather than preventing it. Using that prevents having to use something stronger. If you put something there and prevent pneumonia, that's better than having it come back with whatever, you know, LA-200 or whatever else you're going to use… [No. 13, focus group 1].

For the questionnaire respondents, seven (17.5%) strongly agreed with the statement “The VFD would lead to increased use of injectable antibiotics by producers,” 11 (27.5%) agreed with this statement, 18 (45%) neither disagreed nor agreed, and four producers (10%) disagreed.

### 3.4. Objective 2: Alternatives to Antimicrobials

Most of the dairy producers' alternatives to antimicrobials were geared towards mastitis prevention and control. The focus group participants considered: (a) good management practices; (b) use of vaccines, and immunostimulants; and (c) early disease detection as their alternatives to antimicrobials. The excerpts that support these perceived alternatives are provided below.

*(2a) Good Management Practices*. The husbandry practices considered alternatives to AMU included proper animal nutrition, proper housing, and infection control measures. Specifically, good milking parlor management, clean cow facilities, and good udder health management were reported to be alternatives to AMU. Examples of good udder health management practices mentioned include the use of teat dips, teat sprays, and teat sealants.

…I agree with managing your facilities properly. All your milking equipment and housing and whatever plays a big part in it… [No.13, focus group 1].

…we use teat sealant[s]… [No.9, focus group 2].

*(2b) Vaccines and Use of Immunomodulatory Products*. Vaccinations and use of immunomodulatory products, such as pegbovigrastim (Imrestor®), were frequently mentioned as an alternative to antimicrobials. It was mentioned that immunomodulatory products are used in fresh cows to minimize AMU.

… Well, I started using it [Imrestor®] temporarily just because it's supposed to help these cows, you know, fresh cows and keep the drug use down.… [No.11, focus group 1].

*(2c) Use of Appropriate Technology for Early Disease Detection*. Early disease detection using appropriate technology, such as rumination monitors, was considered important in minimizing and reducing AMU.

… we have a monitoring system that monitors rumination as well as activity. So, when her rumination goes down, you know something's wrong. And maybe you can prevent it or treat it before it gets bad… [No.12, focus group 1].

Additional training for dairy producers on infection prevention and control was supported by many survey respondents. Two participants (5.1%) strongly agreed that infection prevention and control measures (farm-level biosecurity and vaccination) would reduce AMU in dairy operations, 17 respondents (43.6%) agreed, 17 (43.6%) neither disagreed nor agreed, and three (7.7%) strongly disagreed.

### 3.5. Objective 3: Knowledge of AMR, and Perceptions Regarding AMR

Many focus group participants as well as survey participants were familiar with AMR. The salient findings for our third objective are presented below in detail under the themes: (a) knowledge of AMR; (b) perceptions regarding AMR emergence; and (c) proposed solutions to AMR.

*(3a) Knowledge of AMR*. Some focus group participants demonstrated their knowledge of AMR and believed there was “some amount” of AMR occurring in food animal pathogens. Also, the repeated treatment of animals with antimicrobials was mentioned in the discussions.

…As far as antibiotic resistance, there is some out there. I don't think it's gone completely from food animals… [No.5, focus group 1].

…There'd be 25-30 percent chance of a repeat [treatment of animals with antimicrobials] … [No.6, focus group 1].

The extent to which survey questionnaire respondents were familiar with or concerned about AMR varied among the respondents (Table 4). Producer's gender (male vs female; P = 0.699), herd size (P = 0.447), education level (P = 0.524, age (P = 0.508), and number of years in cattle farming (P = 0.535), were not significantly associated with producer's degree of concern about AMR. Based on these findings, no meaningful multivariable analyses could be performed.

*(3b) Perceptions regarding AMR Emergence*. Participants attributed the emergence and occurrence of AMR to the over-use and prolonged use of the same antimicrobials without rotating and the lack of new antimicrobials. The problem of AMR in human pathogens was attributed to antimicrobial over-use in humans and not in livestock.

… [Antimicrobial resistance bites you] eventually if you overuse and use the same thing [antimicrobial] too long. It's the same as pesticides. They only work for so long. Hopefully you can get enough variety to where you can switch from one to another and maintain both… [No.11, focus group 2].

…As humans, we do a lot of stuff that probably amplifies that. Everybody's antibacterial nowadays. You can't sneeze without being doused in it almost… [No.5, focus group 1].

Some participants believed that the human health risks associated with AMU in food animals are not evidence-based and generally perceived their AMU practices to be prudent.

…We realize that there's some amount of resistance to antibiotics. But a lot of the population that has these fears of resistance that aren't science based. And they're the ones that tend to drive regulation with non-science-based opinions on antibiotic resistance. If something is science based and real, hey, I'm all for doing it. Because some people in town think that antibiotics in cows cause them to have resistance and there's no science behind it, I think that's a real problem… [No. 6, focus group 1].

The producers believed the public was misinformed about how and why antimicrobials are used in food animals, and the producers associated the misinformation with a lack of consumer education and milk marketing with buzzwords such as “antibiotic free.”

…I think part of the problem with the public is our milk marketing. This jug of milk says antibiotic and hormone free and this one does not. So, they assume that that one has antibiotics in it, which falls into antibiotics in milk and all this antibiotic resistance and stuff like that when no milk has antibiotics in it. But they just don't know that. They're just not educated… [No. 12, focus group 1].

*(3c) Proposed Solutions to AMR*. The participants suggested: (i) improving antimicrobial drug labels; (ii) additional producer training on prudent AMU; and (iii) development of diagnostic tools for rapid on-farm detection of AMR and on-farm antimicrobial sensitivity testing as measures for improving AMU and containing AMR. A brief description of the suggested measures is given below.

*(3c. i) Improving Antimicrobial Drug Labels*. It was suggested that the dosage rates indicated on antimicrobial drug labels need to be changed to reflect the appropriate dosage rates because current antimicrobial drug labels may not reflect the appropriate drug dosage rates.

… The [antimicrobial] labels need to be labeled for appropriate doses instead of what appropriate doses were 40 years ago. All that information needs to be there on the label, so we know what the appropriate dose is, what the appropriate withdrawal is and what the appropriate bug or disease it's going to take care of in a very concise, easy to read, easy to understand label. That would be a most important change… [No.6, focus group 1].

Also, producers perceived the current antimicrobial labels and information on the antimicrobial package inserts to be very technical and difficult to comprehend and suggested that antimicrobial drug labels and package inserts should be written in nontechnical language to make such information easier for producers to understand. To cater to non-English speaking farm employees (Hispanic/Latino farm workers), it was suggested that antimicrobial drug labels be written in both English and Spanish.

Among survey questionnaire respondents, 13 (33.3%) found antimicrobial labels difficult to understand and interpret, whereas 26 (66.7%) found these labels easy to understand and interpret. Education level was not significantly associated with producer's perception of difficulty to comprehend antimicrobial label instructions (OR = 2.24; 95% CI = 0.563, 8.91; P = 0.253). Of the 39 survey participants who responded to the question on the preferred language for antimicrobial label instructions, only three (8%) preferred these labels to be in both English and Spanish, whereas 36 (92%) preferred antimicrobial drug labels to be in English.

*(3c. ii) Additional Training on Prudent AMU*. Participants suggested that more training for dairy producers on prudent AMU was needed for improving AMU in cattle production. However, continuing professional education for medical practitioners on prudent AMU was suggested in order to reduce nonjudicious AMU in humans.

…I'd like to know more information about it [antimicrobial use]. I'd like to be able to treat the animal one time and get it taken care of. It requires some advanced training. And it's hard to get that sometimes.… [No. 5, focus group 2].

…I have a statement about the human side of it. They need to educate doctors that prescribe all of these liquid antibiotics to children for earaches and everything else when they're not earaches and different things. And I think that's what causes resistance in humans… [unidentified participant, focus group 2].

Additional training for dairy producers on prudent AMU practices was supported by approximately a third of the survey respondents. Four producers (10%) strongly agreed that producers required additional training on prudent AMU, 10 (25%) agreed, 15 (37.5%) neither disagreed nor agreed, nine (22.5%) disagreed, and two (5%) strongly disagreed.

*(3c. iii) Development of Diagnostic Tools for Rapid On-Farm Detection of AMR and On-Farm Antimicrobial Sensitivity Testing*. It was suggested in the focus groups that producers should be able to test cows on-farm for AMR and antimicrobial susceptibility. Such on-farm diagnostics would properly orient antimicrobial therapy and guide the implementation of appropriate on-farm isolation measures.

… [We should be] able to test the cows on the farm – your own antibiotic and your own somatic cell. We had a product that we were getting from RapiDEC for somatic cells. For some reason they took it off the market… Products like that can help us on the farm… [No. 1, focus group 1].

### 3.6. Objective 4: Avenues for Receiving Information on AMU

In the focus groups, participants identified the following as viable avenues for receiving information on prudent AMU: the veterinarian, email, dairy publications, and producer meetings. The producers considered the veterinarian (for areas with food animal vets) to be a trusted source of information on prudent AMU.

…Our vet has a meeting once a year where he will bring in sponsors that will be reps of his companies mail list. It's generally whenever we have a question, we call and ask. He's our source of information… [No. 3, focus group 2].

Regarding avenues/formats for receiving information on prudent AMU, no single medium was most preferred by survey questionnaire respondents. The most commonly mentioned avenues for receiving information on prudent AMU included brochures (n = 8), educational seminars (n = 6), and a producers' handbook on prudent AMU (n = 4). These formats for receiving information were chosen individually or in combination with others, such as AMU flowcharts for the barn, videos on prudent AMU, and laminated posters.

## 4. Discussion

Jaccard's similarity index and the survey participant demographics showed that there was diversity of opinions among participants in the present study. Our study utilizes the strength of a mixed methods research design (a combination of qualitative and quantitative methods) to extend the knowledge of AMU in dairy production by highlighting the diversity and complexity of factors driving AMU among dairy producers in TN. Additionally, we identified the dairy producers' alternatives to antimicrobials, their perceptions regarding AMR, and the appropriate avenues and formats for disseminating information on prudent AMU to these producers. Gussmann et al. suggested that campaign efforts that target improvements in AMU among farmers need to take into account farmers' usual AMU practices in order to motivate farmers to adopt control measures that facilitate prudent AMU [4]. Therefore, our findings should aid in optimizing the efforts under which targeted campaigns for nationwide AMS are applied in US dairy production.

A previous survey by the U.S Department of Agriculture (USDA) found that producers on almost all the sampled dairy operations (99.7%) reported having at least one case of mastitis during 2013 and antimicrobials were administered to mastitic cows on 96.9% of dairy operations [19]. In the present study, mastitis was the most commonly mentioned disease for which antimicrobials were used. This is not surprising because mastitis is known to be the most frequent disease of dairy cows [20]. To minimize AMU, TN dairy producers should be encouraged to strengthen their herd health measures for mastitis prevention and control.

In the Netherlands, the introduction and implementation of the farm health plan and farm-specific treatment protocols contributed to the reduction in AMU [10, 11]. Similarly, in the UK, use of alternative treatment protocols for diseases in which critically important antimicrobials (CIAs) are used led to significant reductions in the use of CIAs [21]. The use of written protocols for treating sick animals with antimicrobials could reduce treatment errors, since most of antimicrobial treatments in farms are often administered by non-technical farm personnel (the farmer or farm employees) [22, 23]. Although the FDA guidance requires producers using prescription antimicrobials to have written treatment protocols developed in collaboration with the herd veterinarian [13], many questionnaire respondents, in the present study, mentioned that their farms did not have written protocols for treating sick animals with antimicrobials. This finding suggests a need for TN veterinarians and dairy extension agents to emphasize and encourage the development and use of written AMU protocols. The establishment and implementation of written treatment protocols could be made a mandatory requirement for all producers in the state, and possibly the entire U.S.

In the present study, a section of the focus group participants self-reported their use of C/S test results for on-farm pathogen surveillance. Similarly, many producers who completed the questionnaire self-reported their use of C/S to determine the causes of disease in their farms and to select antimicrobials for farm use. These findings generally suggest that, although not universally practiced, use of C/S test results for on-farm pathogen surveillance and for antimicrobial selection is a widespread and common practice among TN dairy farmers. Producers not utilizing C/S could be constrained by cost, lack of rapid C/S tests or lack of awareness about the benefits of C/S. A previous European study highlighted the need for cheaper and rapid C/S testing and more education for animal owners about the benefits of C/S [24]. Also, a previous Dutch study found that the financial and labor investments associated with implementation of veterinary advice are the reasons farmers do not comply with veterinary recommendations [25]. These findings on use of C/S are also in contrast to those of a previous New Zealand study, where C/S testing is perceived to be not useful because it did not influence what antimicrobial the veterinarian prescribed and, hence, is not widely used by dairy producers [14]. Possibly, use C/S test results is widespread and common among TN dairy producers because its economic value is appreciated by many producers.

Our findings show that profitability of the dairy operation (economic gain) was a key factor influencing the decisions of many producers to use antibiotics. In their dairies, cows perceived to be economically less valuable were culled, rather than treated. Additionally, the focus groups identified the lactation stage as a factor driving AMU by dairy producers. This association between lactation stage and AMU could be due to high milk yield at peak lactation and changes in immune function at early lactation. The pregnancy status of the cow (in-calf or open) during the lactation period may also be a factor that producers consider when deciding to use antimicrobials. It is possible that these producers treat high milk yielding cows with antimicrobials in case of udder health problems to maintain high economic performance. A Danish study found that high milk yield was associated with a higher probability of both lactational and dry-off antimicrobial treatment of dairy cows [4]. High milk production is a known risk factor for occurrence and recurrence of clinical mastitis, whose occurrence drives AMU [4, 26, 27]. Changes in immune function and nonspecific host defense mechanisms are reported to be associated with high incidence of clinical mastitis in early lactation [26]. To minimize the economic losses associated with intramammary infections, a section of focus group participants mentioned using dry cow therapy as a blanket antimicrobial treatment at their farms to control the risk of new intramammary infections during the dry period. This practice of blanket dry cow therapy is concerning and suggests a need for veterinarians and dairy extension agents to encourage TN dairy producers to avoid blanket dry cow therapy and adopt selective dry cow therapy to minimize unnecessary AMU. Although still a common practice in the US, blanket dry cow therapy is now illegal in several European countries to avoid selection for AMR [4, 28, 29]. Similarly, previous studies have shown that blanket dry cow therapy may not be an optimal approach to dry cow therapy when compared to selective dry cow therapy, and dry cow therapy does not compromise animal welfare and productivity and is economically more beneficial compared to blanket dry cow therapy [29–32]. A policy shift towards banning blanket dry cow therapy in TN and the entire US may be worth exploring.

Our findings showed that veterinarian recommendations and peer recommendations generally influence AMU practices of dairy producers. Additionally, we identified the veterinarian, producer meetings, and educational seminars (along with other avenues) to be viable ways for reaching out to producers. Similar to other research [16], our findings suggest that veterinarians and peers could act as agents of change towards prudent AMU among dairy producers. Policy interventions towards prudent AMU should channel AMU-related behavioral change messages to dairy producers through veterinarians (where possible) and other producers (peers) using the identified avenues/formats. Furthermore, targeted behavioral change messages towards prudent AMU practices should be integrated into routine veterinary farm visits and master dairy training programs. Behavioral techniques, such as motivational interviewing informed by assessing producers' readiness for change, could be used [33]. Additionally, a participatory policy making approach with groups of dairy producers could be used to develop antimicrobial stewardship policies as was piloted with dairy farmers in the UK [34]. Producer meetings/associations and educational seminars for producers should be used to identify AMU training needs and raise more awareness about AMR and prudent AMU among dairy producers.

The VFD was mentioned to have limited access to preventive in-feed antimicrobials (e.g., Aureo S 700®), and as a result, is believed to be driving increased use of injectable antimicrobial agents. Aureo S 700® contains 3 antimicrobials (aureomycin, chlortetracycline, and sulfamethazine) and is indicated for the use of weight gain maintenance and the management of stressful conditions in calves. We did not ascertain, in the present study, if the increased use of injectable antimicrobial agents was for prophylactic and/or therapeutic purposes. We suggest a nation-wide investigation of the impact of the VFD on the use of injectable antimicrobials among US dairy producers be conducted.

Although 12 survey participants reported to be very familiar with AMR, a considerably large number (21) were moderately familiar, while others were either slightly familiar or not familiar at all. Similarly, it is concerning that 10 (22.73%) reported they were not concerned about AMR, and 3 producers (6.82%) did not rate their degree of concern about AMR because they were not familiar with what AMR meant. These findings suggest a need for more sensitization of producers on AMR and AMU.

Researchers in Australia suggested that veterinary antimicrobial drug labels need regular updating to reflect the appropriate dosage rates for treatment of common veterinary pathogens [35]. To improve AMU, our focus group participants suggested that antimicrobial dosage rates indicated on certain antimicrobial drug labels need to be changed to reflect the appropriate dosage rates. A targeted study evaluating the appropriateness of dosage rates indicated on drug labels for currently used veterinary antimicrobials in the US is necessary to validate or dispute this finding. A previous study conducted in South Carolina reported that the dairy industry often relies on Hispanic labor, and the language barrier was a challenge when dealing with non-English speaking farm employees [16]. In the present study, a section of focus group participants suggested that antimicrobial drug labels should be in both English and Spanish to cater for non-English speaking farm employees (Hispanic/Latino farm workers), and only three (7.69% [3/39]) producers who responded to the questionnaire preferred antimicrobial drug labels to be in both English and Spanish. Possibly, these three questionnaire respondents who preferred antimicrobial drug labels to be in both English and Spanish utilize Hispanic labor in their dairy farms. Additionally, a section of focus group participants and a third of the dairy producers (33.33%, (13/39)) who completed the questionnaire perceived the current antimicrobial labels and information on the antimicrobial package inserts to be very technical and difficult to comprehend. Our findings showed that producers' education levels were not significantly associated with producers' perceptions of difficulty to comprehend antimicrobial label instructions, perhaps due to the few survey respondents. There is need to conduct a country-wide investigation of this perception that current antimicrobial labels and information on the antimicrobial package inserts are very technical and difficult for producers to comprehend. Friedman et al., based on their South Carolina study, recommend that all farm health resources and interventions should be bilingual (in English and Spanish) and in an easy-to-understand language to cater to the growing population of Hispanic/Latino farm employees [16]. As suggested by the producers during the focus group discussions, we contend that there is a need for US veterinary pharmaceutical companies to consider labeling antimicrobial drugs in both English and Spanish and in nontechnical language for easier comprehension.

Globally, there is increased debate about the agricultural use of antimicrobials and its contribution to AMR and this has created the desire for “antibiotic-free” meat products among some consumers [36]. As a result of this increased public scrutiny of AMU in animal agriculture, livestock producers are under pressure to maintain consumer confidence in their products [37]. In the present study, the producers believed that the public was misinformed about AMU in food animals and associated this misinformation with a lack of consumer education, suggesting a need to create more public awareness regarding how and why antimicrobials are used in food animals.

In qualitative studies such as focus groups, the presence of researchers during data gathering may affect responses [38], leading to social desirability bias [39]. Social desirability bias may also be an issue in survey studies [40]. Our focus groups and survey participants could have given socially desirable responses, thus introducing bias to our findings. However, socially desirable responses, if any, could be very minimal, since both focus groups and survey respondents were assured that the data collected was anonymized and participation was voluntary. Prior to the study, there was no established relationship and interaction between any of the researchers and any of the focus group participants that could have influenced participants' responses. Additionally, the survey questionnaire (both paper and online) was self-administered. Thus, participants are likely to have given their true opinions, perceptions, and practices. It is common for studies utilizing focus groups to be biased by the presence of dominant participants. However, in the present study, such bias could be very minimal, if any, because our focus groups were moderated by one of the authors (EBS) with a background in the behavioral/social sciences and wide experience in moderating such meetings.

## 5. Conclusions

There is a need for TN veterinarians and dairy extension agents to emphasize and encourage the development and use of written AMU protocols. The use of these protocols should be mandatory for all dairy producers in the US Use of culture and sensitivity test results for on-farm pathogen surveillance and for antimicrobial selection is a widespread and common practice among TN dairy farmers. There is a need for more awareness about C/S to encourage producers not utilizing it to adopt its use. Blanket dry cow therapy is still commonly practiced by some dairy producers in TN. There is need to popularize/promote selective dry cow therapy and its associated benefits among dairy producers in the state. An investigation of the impact of the VFD on the use of injectable antimicrobials among US dairy producers should be conducted. Continuing training on prudent AMU is needed for TN dairy producers.

## Figures and Tables

**Table 1:** Demographics of Tennessee dairy producers surveyed concerning antimicrobial use practices, 2017.

**Variable**	**Number (**%**) of respondents**
**Gender **	**n = 39**
Female	7 (18)
Male	31 (79.5)
Preferred not to report gender	1 (2.6)
**Age group (years)**	**n = 37**
20 – 29	2 (5.4)
30 – 39	6 (16.2)
40 – 49	8 (21.6)
50 – 59	13 (35.1)
60 – 69	8 (21.6)
**Education level**	**n = 37**
High school	16 (43.2)
Vocational	2 (5.4)
College	18 (48.7)
Professional	1 (2.7)
**Years in dairy cattle production**	**n = 38**
< 5	1 (2.6)
6 – 10	6 (15.8)
16 – 20	1 (2.6)
21 – 25	4 (10.5)
26 – 30	4 (10.5)
> 30	22 (57.9)
**Herd size**	**n = 37**
1 – 49	2 (5.4)
50 – 99	8 (21.6)
100 – 149	7 (18.9)
150 – 199	5 (13.5)
200 – 299	7 (18.9)
300 – 399	3 (8.1)
400 – 499	1 (2.7)
500+	4 (10.8)
**Raised on a cattle farm **	**n = 39**
Yes	2 (5.1)
No	37 (94.9)

**Table 2:** Survey results showing the practices of Tennessee dairy producers related to antimicrobial use, 2018.

**Practice**	**Number of participants (percentage)**		
	**Yes**	**Not sure**	**No**
Farm keeps up-to-date written records of antimicrobial drug purchases (n = 40)	23 (57.5)	5 (12.5)	12 (30)
Farm keeps written records of medicated feeds purchased in the framework of VFD (n = 40)	20 (50)	3 (7.5)	17 (42.5)
Farm keeps up-to-date written records of antimicrobial drugs used to treat animals (n = 40)	28 (70)	4 (10)	8 (20)
Cattle on the farm are sometimes treated with antimicrobials at dosages higher than the label provision (n = 40)	9 (22.5)	1 (2.5)	30 (75)
Farm practices extra-label AMU (n = 38)	7 (18.4)	2 (5.3)	29 (76.3)
Farm has written protocols for treating sick animals with antimicrobials (n = 38)	17 (44.7)	3 (7.9)	18 (47.4)

**Table 3:** Dairy producers' self-reported use of bacterial culture and C/S in Tennessee dairy farms, 2018.

	**Number of participants (percentage)**				
	Always	Most of the time	About half of the time	Sometimes	Never

Used bacterial cultures to determine cause of disease on their farms (n = 44)	0 (0)	6 (13.6%)	4 (9.1)	26 (59.1%)	8 (18.2)

Used C/S in selection of antimicrobials (n = 43)	1 (2.3)	5 (11.6)	4 (9.3)	25 (58.1)	8 (18.6)

**Table 4:** Questionnaire respondents views on a series of statements related to AMR.

**Number of participants (percentage)**					
	Extremely familiar	Very familiar	Moderately familiar	Slightly familiar	Not familiar at all.

How familiar are you with the subject of antibiotic resistance? (n = 43)	1 (2.3)	12 (27.9)	21 (48.8)	6 (14)	3 (7)

	Very concerned	Moderately concerned	Not concerned	Did not rate their degree of concern about AMR due to their unfamiliarity with the meaning of AMR.	

How do you rate your degree of concern about AMR infections in cattle production? (n = 44)	4 (9.1)	27 (61.4)	10 (22.7)	3 (6.8)	

	Strongly agreed	Agreed	Neither disagreed nor agreed	Disagreed	Strongly disagreed.

Some antibiotics you use on your cattle have become ineffective (there is resistance to antibiotics used in cattle) (n =40)	1 (2.5)	17 (42.5)	16 (40)	5 (12.5)	1 (2.5)

Antibiotic drugs work less effectively than in the past (n = 40)	1 (2.5)	10 (25)	20 (50)	7 (17.5)	2 (5)

## Data Availability

Focus group transcripts pertaining to the manuscript can be obtained from the corresponding author upon reasonable request. The survey raw data used to support the findings of this study are included within the supplementary information files.

## Supplementary Materials

Supplementary 1 S1: Semistructured interview guide.

Supplementary 2 S2: Survey questionnaire.

Supplementary 3 S3: Consolidated criteria for reporting qualitative studies (COREQ): 32-item checklist.

Supplementary 4 S4: Survey raw data.
