# Supplementary material for: Drivers of Antimicrobial Use Practices among Tennessee Dairy Cattle Producers
Source: Vet Med Int. 2018 Dec 27;2018:1836836. doi: 10.1155/2018/1836836 (PMC6327273; doi:10.1155/2018/1836836)
Supplement: Supplementary 3 — S3: Consolidated criteria for reporting qualitative studies (COREQ): 32-item checklist. [file 1836836.f3.docx]

**Consolidated criteria for reporting qualitative studies (COREQ): 32-item checklist**

| **No** | **Item** | **Guide questions/description** |  | **Section where found** |
| --- | --- | --- | --- | --- |
| **Domain 1: Research team and reflexivity** | | | |  |
| **Personal characteristics** | | | |  |
| 1 | Interviewer/facilitator | Which author/s conducted the interview or focus group? | All the 4 authors attended both focus groups. The third author (EBS) moderated the focus group discussions. | Materials and methods. |
| 2 | Credentials | What were the researchers’ credentials? | 1^st^ author (JEE): BVM, MVM, PgDip, PhD Candidate  2^nd^ author (MC): BS, DVM, PhD, DACVIM  3^rd^ author (EBS): BA, MSSW, PhD  4^th^ author (CCO): DVM, MS, PhD, DACVPM (Epi) | N/A |
| 3 | Occupation | What was their occupation at the time of study? | JEE: Graduate Research Assistant/PhD Candidate.  MC: Assistant Professor, Large Animal Clinical Sciences.  EBS: Director Veterinary Social work/ Clinical Associate Professor.  CCO: Assistant Professor, Epidemiology and Food safety. | N/A |
| 4 | Gender | Was the researcher male or female | Male: JEE, MC, CCO  Female: EBS | N/A |
| 5 | Experience and training | What experience did the researcher have? | JEE: Underwent qualitative research methods training while at graduate school and has experience in veterinary clinical practice, teaching senior veterinary students at a veterinary school.  CM: Has extensive experience in food animal veterinary practice.  EBS: Has wide experience in moderating group meetings.  CCO: Has wide experience in epidemiology and food safety. | N/A for JEE, MC and CCO.  The experience of the moderator (EBS) is mentioned in the discussion. |
| **Relationship with participants** | | | |  |
| 6 | Relationship established | Was a relationship established prior to study commencement | There was no established relationship with the participants prior to the study commencement. | N/A |
| 7 | Participant knowledge of the interviewer | What did the participants know about the researcher? e.g. personal goals, reasons for doing the research | The participants knew nothing about the researchers prior to the meetings. However, at the beginning of each focus group discussion, participants were informed about the purpose of the study as part of obtaining an informed consent prior to commencing with the discussions. | N/A |
| 8 | Interviewer characteristics | What characteristics were reported about the interviewer/facilitator? E.g. bias, assumptions, reasons and interests in the research topic. | The participants were informed that the moderator was a non-veterinarian with a background in social work. | N/A |
| Domain 2: Study design | | | |  |
| Theoretical framework | | | |  |
| 9 | Methodological orientation and theory | What methodological orientation was stated to underpin the study? e.g. grounded theory, discourse analysis, ethnography, phenomenology, content analysis | An inductive approach to thematic analysis was utilized. | Materials and methods. |
| Participant selection | | | |  |
| 10 | Sampling | How were participants selected? E.g. purposive, convenience, consecutive, snowball | Participants were purposively selected and participation in the study was voluntary. | Materials and methods. |
| 11 | Method of approach | How were participants approached? E.g. face-to-face, telephone interview, mail, email | Drs. Liz Eckelkamp and Peter D. Krawczel of the Department of Animal Science at the University of Tennessee and Mr. Stan Butt of the Tennessee Dairy Producers Association helped with the mobilization of participants and organizing the dairy focus groups. | Acknowledgements section |
| 12 | Sample size | How many participants were in the study? | Twenty three (23) producers participated in the focus groups. | Materials and methods. |
| 13 | Non-participation | How many people refused to participate or dropped out? Reasons? | No participant dropped out of the focus groups. | N/A |
| Setting | | | |  |
| 14 | Setting of data collection | Where was the data collected? Home, clinic, workplace? | We collected the data at the county extension center and at a local restaurant were the focus groups were held. | Materials and methods. |
| 15 | Presence of non-participants | Was anyone else present besides the participants and researchers? | No | N/A |
| 16 | Description of sample | What are the important characteristics of the sample? e.g. demographic data, date | Focus group 1 had one female and 11 male participants, while focus group 2 had two females and nine male participants. | Results section: Focus group participant characteristics. |
| Data collection | | | |  |
| 17 | Interview guide | Were questions, prompts, guides provided by the authors? Was it pilot tested? | Yes. Questions, prompts, guides were provided. The interview guide was provided. There was no specific separate pilot testing done. However, the interview guide was modified based on participant comments after the first focus group. | Materials and methods. |
| 18 | Repeat interviews | Were repeat interviews carried out? If yes, how many? | Repeat interviews were not carried out. | N/A |
| 19 | Audio/video recording | Did the researchers use audio or visual recording to collect the data? | Data was video recorded. | Materials and methods. |
| 20 | Field notes | Were field notes made during and/or after the interview or focus group? | Yes | Materials and methods. |
| 21 | Duration | What was the duration of the interviews or focus groups? | The focus groups lasted approximately 60 minutes. | Materials and methods. |
| 22 | Data saturation | Was data saturation discussed? | We could not determine if data saturation was reached during the second focus group discussion. | Materials and methods. |
| 23 | Transcripts returned | Were transcripts returned to participants for comment and/ or correction? | No. Participants could not be identified because data was de-identified at collection for protection of human subjects in research. | N/A |
| Domain 3: Analysis and findings | | | |  |
| Data analysis | | | |  |
|  | Number of data coders | How many data coders coded the data | All the four authors coded the data | Materials and methods: data analysis section. |
| 25 | Description of the of the coding tree | Did authors provide a description of the coding tree? | The coding is described in the manuscript. | Materials and methods: data analysis section. |
| 26 | Derivation of themes | Were themes identified in advance or derived from the data? | Themes were not identified in advance. Final themes presented in the manuscript were arrived at after a review & harmonization meeting to compare individual data coding. | Materials and methods: data analysis section. |
| 27 | Software | What software, if applicable, was used to manage the data? | NVivo qualitative data analysis Software; QSR International Pty Ltd. Version 12, 2018 was used. | Materials and methods: data analysis section. |
| 28 | Participant checking | Did participants provide feedback on the findings? | No. Participants were de-identified, hence could not be traced back. | N/A |
| Reporting | | | |  |
| 29 | Quotations presented | Were participant quotations presented to illustrate the themes/findings? Was each quotation identified e.g. participant number | Yes, quotations from different participants were presented verbatim (in participants’ own words) to illustrate the themes/findings. Each quotation was identified by participant number. | Results section |
| 30 | Data and findings consistent | Was there consistency between the data presented and the findings? | Yes | N/A |
| 31 | Clarity of major themes | Were major themes clearly presented in the findings? | Yes | Results section. |
| 32 | Clarity of minor themes | Is there a description of diverse cases or discussion of minor themes? | Yes | Results section. |
